# The first complete chloroplast genome sequence of *Lanxangia tsaoko* and phylogenetic analysis

**DOI:** 10.1080/23802359.2019.1629354

**Published:** 2019-07-11

**Authors:** Jing Meng, Hui Jiang, Jun He, Yonghong He, Yuwen Zhang, Yan Zhao

**Affiliations:** a College of Horticulture and Landscape, Yunnan Agricultural University, Kunming, China;; b Kunming Institute of Botany, Chinese Academy of Sciences, Kunming, China;; c College of Plant Protection, Yunnan Agricultural University, Kunming, China

**Keywords:** *Lanxangia tsaoko*, complete chloroplast genome, phylogenetic analysis, traditional Chinese medicine

## Abstract

*Lanxangia tsaoko* is a famous Chinese traditional medicine with long history. In this study, the complete chloroplast (cp) genome of *L. tsaoko* was characterized and assembled using a high-throughput sequencing method. The complete cp genome is 164,008 bp in length, including a large single copy (LSC) region of 89,134 bp and a small single copy (SSC) region of 15,372 bp separated by two inverted repeat (IR) regions of 29,751 bp. A total of 137 genes were identified, including 88 protein-coding genes (PCG), 38 transfer RNA genes, 8 ribosomal RNA genes, and 3 pseudogenes. The phylogenetic analysis revealed that *L. tsaoko* is located in Zingiberaceace, closely related to *Amomum* and *Alpinia* with a 100% bootstrap support.

*Lanxangia tsaoko* (Crevost & Lemarié) M. F. Newman & Škorničk. (Zingiberaceae) is a perennial herb of *Lanxangia* M. F. Newman & Škorničk., which is geographically restricted to southern China (Yunnan), northern Laos, and Vietnam (Boer et al. 2018). It was once one of the members of *Amomum* Roxb. according to morphological characters in the past, but has long been challenged. Combined with the molecular data and morphological characters, new genera *Lanxangia* was described and separated from *Amomum* (Boer et al. 2018). *Lanxangia tsaoko* has been used for centuries as food, spice, and perfume in China, Japan, and Korea. Its fruit has been used in traditional Chinese medicine to treat inflammatory and infectious diseases, such as throat infections, malaria, abdominal pain, and diarrhea. Phytochemicals have been reported, such as bicyclononane, tsaokoin, isotsaokoin, diarylheptanoids, and so no (Song et al. 2001; Moon et al. 2004; Starkenmann et al. 2007; Lee et al. 2008). To better understand genetics information for further development and utilization strategy, we reported and characterized the complete cp genome of *L. tsaoko* (GenBank Accession Number: MK937808).

Fresh leaves of *L. tsaoko* were collected from Puer (Yunnan, China; 28°49′53″ N, 100°58′23″ E). Voucher specimen was deposited in the Herbarium of KUN. Total genomic DNA was extracted using modified CTAB method (Doyle and Doyle 1987). Reads of the complete cp genome were assembled using CLC Genomic Workbench v10 (CLC Bio., Aarhus, Denmark). All the contigs were checked against the reference genome of *Amomum compactum* (MG000589) using BLAST (https://blast.ncbi.nlm.nih.gov/) and aligned contigs were oriented according to the reference genome. The complete cp genomes were then constructed using Geneious v4.8.5 (Biomatters Ltd., Auckland, New Zealand) and was automatically annotated using DOGMA (http://dogma.ccbb.utexas.edu/). To identify the phylogenetic position of *L. tsaoko*, the maximum likelihood (ML) tree was conducted by MEGA v7.0 (Kumar et al. 2016) with 1000 bootstrap replicates based on the alignments created by the online program MAFFT (https://mafft.cbrc.jp/alignment/server/index/index.html) using already published complete cp genomes.

The complete cp genome of *L. tsaoko* is 164,008 bp in length, comprising of a large single copy (LSC) region of 89,134 bp and a small single copy (SSC) region of 15,372 bp, separated by two inverted repeat regions (IRs) of 29,751 bp. The overall GC content is 36.0%. The genome contained total of 137 genes, including 88 protein coding genes, 38 tRNA genes, 8 rRNA genes, and 3 pseudogenes.

To investigate the phylogenetic position of *L. tsaoko*, 9 other published complete cp genomes of Zingiberaceae were used to construct a phylogenetic tree, using *Costus pulverulentus* (KF601573) in Costaceae and *Canna indica* (KF601570) in Cannaceae as outgroups. The phylogenetic analysis revealed that *L. tsaoko* is located in Zingiberaceace, closely related to *Amomum* and *Alpinia* with a 100% bootstrap support, which was in accordance with the previously result (Figure 1) (Boer et al. 2018). This complete cp genome can be subsequently used for phylogenetic and genetic engineering studies of *L. tsaoko*, and would be fundamental to formulate potential development and management strategies for this important medicine herb.

**Figure 1.**
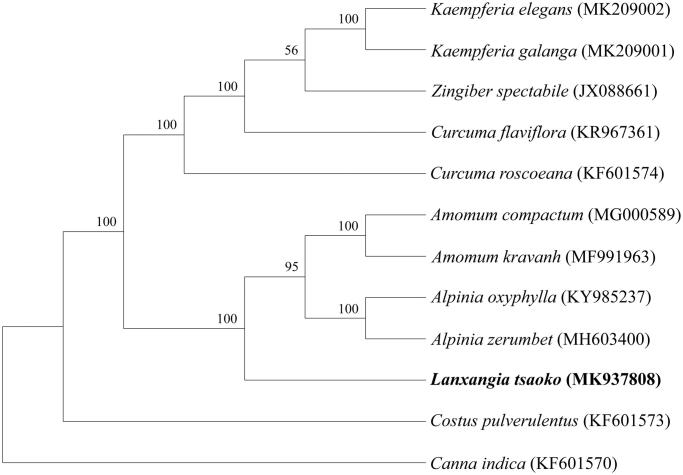
Phylogenetic analysis of 12 species based on maximum likelihood (ML) of the complete chloroplast genome sequences. Bootstrap support values (%) are indicated on each node.
